# The management of complicated glaucoma

**DOI:** 10.4103/0301-4738.73686

**Published:** 2011-01

**Authors:** C I Clement, Ivan Goldberg

**Affiliations:** 1 Glaucoma Unit, Sydney Hospital and Sydney Eye Hospital, Macquarie Street, Sydney NSW Australia 2000; 2 Central Clinical School, The University of Sydney, Sydney NSW Australia 2006; 3 Save Sight Institute, Macquarie Street, Sydney NSW Australia 2000; 4 Eye Associates, Level 4, 187 Macquarie Street, Sydney NSW Australia 2000

**Keywords:** Angle-recession, intraocular pressure, keratoplasty, rubeosis, silicone oil, steroids, uveitis, vitrectomy

## Abstract

Complicated glaucomas present considerable diagnostic and management challenges. Response to treatment can be unpredictable or reduced compared with other glaucomas. However, target intraocular pressure and preservation of vision may be achieved with selected medical, laser and surgical treatment. The evidence for such treatment is expanding and consequently affords clinicians a better understanding of established and novel techniques. Herein we review the mechanisms involved in the development of complicated glaucoma and the current evidence supporting its management.

Complicated glaucomas are difficult to manage. Most often, they are secondary and may be characterized by a mixture of very high intraocular pressure (IOP), multiple and possibly changing mechanisms for raised IOP, ocular factors limiting assessment and/or treatment, often the need for combined therapy and the potential for rapid deterioration in vision.

IOP may be reduced by medical, laser and/or surgical means. The chances for success are maximized when the cause for raised IOP is identified and able to be treated. In some instances, certain treatment options are not effective or are contraindicated.

## Basic Treatment Principles

### Medical reduction of IOP

Initial treatment is usually medical, with the aim being either long-term control or a “safer” IOP whilst awaiting more definitive treatment. The same agents are available as for other glaucomas, but have been less extensively studied for this purpose. Whilst the efficacy and safety of the prostaglandin analogues and beta-blockers in primary open-angle glaucoma (POAG) are well known, this does not apply equally to complicated glaucomas. Response to medical treatment may be unpredictable or reduced, with a few notable exceptions.

In conditions where there is pre- or post-trabecular pathology causing elevated IOP, medical treatment on average appears less effective. Retrospective analysis of patients with glaucoma secondary to iridocorneal endothelial syndrome suggests a poorer response to medical treatment,[1] with 88% requiring filtration surgery. Similarly, efficacy of prostaglandin analogues is reported to be less in pediatric glaucomas[2] and glaucoma associated with Sturge-Weber syndrome.[3,4] There are little data on the IOP-lowering potency of medical agents in rubeotic glaucoma, although it is considered to be poor.

More favorable responses to medical treatment have been reported where there is reduced outflow facility from pathology within the trabecular meshwork despite an open angle. Scherer and Hauber[5] reported a 28% reduction in steroid induced IOP with prostaglandins, comparable with the response seen in POAG. Prostaglandins may be more effective than beta-blockers at lowering and stabilizing IOP in secondary glaucomas with pigment dispersion[6] and at least as effective in many cases of uveitic glaucoma (*vide infra*). An exception is inflammatory glaucoma associated with juvenile arthritis, where IOP is difficult to control medically.[7]

## Laser and/or surgical reduction of IOP

When IOP cannot be controlled by medical means alone, laser and/or surgical options may be considered; there is no consensus on the best approach.

The least invasive option remains laser trabeculoplasty (LT) but its application is limited in this setting. Whilst predominantly used in POAG and ocular hypertension, the response to LT has been reported in steroid-induced glaucoma, pseudoexfoliation glaucoma (PXFG), pigment dispersion glaucoma (PDG) and glaucoma associated with penetrating keratoplasty (PK). Control of steroid-induced glaucoma has been reported[8] and response in PXFG is similar to that achieved in POAG with an average IOP reduction of 31.4%, 18 months after treatment.[9] Paradoxically, elevated IOP has been reported in PDG following LT.[10,11] In PK-associated glaucoma, a 29.7% reduction in IOP was maintained for 22 months following argon LT (ALT).[12] However, LT in this group is often difficult due to opaque cornea, scarring at the host-graft interface or narrowing of the iridocorneal angle.

The commonest surgical options include trabeculectomy (with or without antimetabolites) or glaucoma drainage devices (GDD). Most glaucoma surgeons still prefer trabeculectomy over GDD in most complicated glaucomas, although GDD use is increasing.[13] This is paralleled by a decline in the rate of antimetabolite use with trabeculectomy despite i) a high level of use (68-83%) for “routine” trabeculectomy and ii) evidence that outcome is enhanced with antimetabolite use in complicated cases.[14,15] The reason for this shift is not clear but may relate to concern over increased rates of late postoperative complications such as bleb leak and blebitis in cases where antimetabolites have been used. For example, a mitomycin-C (MMC) trabeculectomy in uveitic glaucoma has an odds ratio for an avascular bleb of 3.93 compared with the rate in eyes with POAG normal tension glaucoma (NTG).[16] Similarly, retrospective analysis of complication rates after trabeculectomy with antimetabolite for uveitic glaucoma found serous choroidal detachment (21%), shallow or flat anterior chamber (AC) (16%), hypotony maculopathy (7%), “malignant” glaucoma (2%), cystoid macular edema (2%) and late-onset bleb leak (2%).[17] On the other hand, a prospective study of antimetabolite augmented trabeculectomy in neovascular glaucoma found a high rate of early postoperative hyphema (88%) but no cases of bleb leak, blebitis, hypotony or suprachoroidal hemorrhage up to 60 months post surgery.[18] Unsurprisingly, practice is divided among glaucoma specialists given such conflicting evidence. Further prospective research with longer follow-up is needed.

The reluctance to use antimetabolites in a population with increased risk of bleb failure may explain the trend toward GDDs to lower IOP. GDDs have had variable success in a number of conditions including neovascular glaucoma (22–78% controlled),[19–24] uveitic glaucoma (75–100% controlled)[20,21,25,26] and congenital glaucoma (44–95% controlled).[19–21,27–31] The IOP lowering benefit of GDD surgery in complicated glaucoma is comparable with that of trabeculectomy alone,[32,33] but with lower rates of hypotony, surgical failure and re-operation. Perhaps as experience with GDD surgery and the supporting literature expands, its use as primary surgical treatment will become more popular.

Cycloablation is an effective way to reduce IOP. Currently available techniques include diode laser, YAG laser or cryotherapy. Diode ablation seems safest with a lower reported incidence of postoperative hypotony.[34] However, visual deterioration is a common complication (5–55%),[34,35] most often from progressive diabetic retinopathy or corneal decompensation. Therefore, this treatment is often reserved for cases refractory to other therapies or with poor visual potential. The mean IOP lowering effect ranges from 26 to 56.3%[34–36] and is maintained for up to 66 months following the procedure. Although complete withdrawal of treatment is a possibility following cyclodiode photoabalation, it is more realistic to achieve withdrawal of systemic treatment (such as acetazolamide) with or without reduction of topical medication.[34] Endoscopic cyclophotoablation (ECP) is claimed to be a safe and effective variation,[37] but requires expertise and specialized equipment and is not possible in the phakic patient. At least one good quality prospective study has shown ECP to be equal to GDD in control of IOP in patients who are pseudophakic and with glaucoma refractory to prior trabeculectomy.[38] Importantly, there were fewer complications in the ECP group.

Non-penetrating glaucoma surgery (NPGS) might be a surgical option for managing complicated glaucomas. Viscocanalostomy augmented with antimetabolite (MMC) has a reported success rate of 85% after 4 years in eyes at risk of trabeculectomy failure.[39] However, the exclusion of patients with uveitis, trabeculitis, irido corneal endothelial (ICE) syndrome or peripheral anterior synechiae (PAS) from any other cause diminishes the relevance of these findings for complicated glaucomas. Further, this technique is more challenging than other IOP-lowering procedures, with a steep learning curve; this will slow any uptake of this procedure.

## Targeted Therapy

### Steroid-induced glaucoma

Widely used following ocular surgery as well as to treat several ophthalmic conditions (including corneal disease, uveitis and retinal edema), corticosteroids are delivered topically for anterior segment disease and peri-ocularly or with intraocular depots for posterior segment conditions. A common complication for both modes of delivery is raised IOP.

First, reduce or withdraw the steroid. As not all topical steroids have equal effects on IOP,[40] it may be possible to switch to a different topical steroid rather than withdraw altogether. In individuals manifesting dexamethasone ocular hypertensive response, fluorometholone may allow better IOP control,[41,42] and the aqueous concentrations of steroid required for anti-inflammatory control may be suboptimal,[43] leading the inflammation to recur.

Substitution of topical steroid treatment may be possible; postoperatively, a non-steroidal anti-inflammatory may be beneficial. Diclofenac may offer superior control of ocular inflammation and reduced risk of postoperative macular edema compared with betamethasone following small incision cataract surgery.[44] Similarly, replacement of a topical steroid with cyclosporine A has been used in individuals whose IOP increases secondary to steroids following PK.[45,46] In both studies, cyclosporine A therapy was associated with a significant reduction in IOP whilst maintaining graft clarity. However, risk of graft rejection increased with cyclosporine A.

Where topical steroid treatment must continue, topical ocular anti-hypertensive medication(s) might help. The commonest agents used are β-blockers as monotherapy or in combination with other classes of agents. Prostaglandin analogues are used less frequently as monotherapy despite evidence suggesting good efficacy[5] because of concerns over their potential effects on the blood–aqueous barrier and increased ocular inflammation (*vide infra*).

The frequency of IOP > 21 mmHg after intravitreal triamcinolone (IVTA) can be as high as 40%, 9 months after initial injection.[47,48] Medical therapy is often enough to maintain IOP within an acceptable range, but occasionally trabeculectomy or a GDD is required.

Selective laser trabeculoplasty (SLT) shows benefit as an alternative to medical therapy or in addition to it, in management of steroid-induced glaucoma.

### Glaucoma in uveitis

Raised IOP in uveitis is common. Up to 40%[49–51] of patients with uveitis develop raised IOP at some time during the course of the condition or its treatment. While patients with chronic uveitis are more likely to have raised IOP than patients with acute uveitis, the primary location of the uveal inflammation (anterior versus posterior) appears to be less important.[50] Although the commonest reason for raised IOP is steroid use,[51] other factors may contribute including inflammation (increased aqueous viscosity, trabeculitis) and secondary structural changes (PAS, iris and angle rubeosis, posterior synechiae with iris bombe, forward rotation of the ciliary body/lens diaphragm).

Aside from treating inflammation, initial treatment for raised IOP in uveitis is usually with topical therapy. Prostaglandins have been avoided or used cautiously due to concerns that they decrease the blood-aqueous barrier with increased intraocular inflammation and cystoid macular edema. Support for this caution comes from animal studies, case reports and retrospective case series.[52] However, some commentators question the scientific validity of avoiding prostaglandin use in uveitis[53] and recent quality research backs this position. A randomized prospective trial[54] and two large independent retrospective case series[55,56] demonstrated i) no association between prostaglandin analogue use and the development of cystoid macular edema or increased ocular inflammation in uveitis and ii) significant reduction in IOP following initiation of prostaglandin analogues. On balance, current evidence supports prostaglandin use.

IOP may be elevated by angle-closure secondary to posterior synechiae and iris bombe. Peripheral laser iridotomy (PLI) is advocated as initial treatment in this setting but has a much higher failure rate (60%) than seen in primary angle closure.[57]

Techniques to promote PLI success in this setting include creating multiple PLIs, making the PLIs larger, aggressive use of anti-inflammatories and possible surgical iridectomy.

Where medical therapy has failed to control IOP, optic nerve damage is occurring, or is at great risk of occurring, and there is no pupil block, trabeculectomy is indicated. Ideally, this should be performed in a non-inflamed eye, although this is rarely the case and explains the higher failure rate. Success may be increased by the use of antimetabolites although they appear to be used less often compared with “routine” glaucoma. Despite using antimetabolites, uveitic eyes are still more likely to require postoperative 5-fluorouracil (5-FU) injections and topical IOP lowering treatments compared with non-uveitic glaucoma.[58,59] The use of a GDD may offer better IOP control in uveitis than does trabeculectomy not augmented by antifibrotics.[30]

### Glaucoma secondary to neovascularization

Neovascularization most commonly follows proliferative diabetic retinopathy or ischemic central retinal vein occlusion. Other causes include ocular ischemic syndrome, central retinal artery occlusion, chronic uveitis, longstanding retinal detachment, radiation retinopathy and ocular tumors. The process involves proliferation of a fibrovascular membrane across the iris and iridocorneal angle, leading to secondary angle closure and reduced outflow facility. Prognosis is usually poor.

Anti-vascular endothelial growth factor (anti-VEGF) agents offer promise in the treatment of neovascular glaucoma. They decrease neovascularization, lower IOP (and thereby improve vision) by halting PAS formation. Both case reports[60,61] and small prospective case series[62,63] of intravitreal bevacizumab (Avastin) have been consistent. In the largest case series so far,[23,62] patients received three injections at 4-weekly intervals with follow-up for 12 months. By the third injection, there had been regression of iris neovascularization in most cases with mean Snellen Visual Acuity (VA) improvement from Counting Fingers to 20/50 and a mean reduction in IOP of 13 mmHg. Three eyes required a GDD to control IOP and 15 eyes remained on at least two topical IOP lowering agents (timolol–dorzolamide). The largest reductions in IOP and improvement in VA occur in those with highest IOP and worst VA at presentation.

Pan-retinal photocoagulation remains central to the long-term control of rubeosis. Patients who received pan-retinal photocoagulation with or without combined bevacizumab use have been compared retrospectively.[64] Those receiving bevacizumab achieved better IOP control throughout follow-up, used less topical IOP lowering medications, retained better VA and were less likely to need GDD to control IOP.

### Glaucoma and silicone oil retinal tamponade

In cases of complex retinal detachment (tractional diabetic retinal detachment, giant retinal tear, proliferative vitreoretinopathy), retinal tamponade is required and is most frequently achieved with silicone oil. Silicone oil leads to a 40% incidence of postoperative glaucoma.[65] Mechanisms for raised IOP may include pre-existing glaucoma, neovascular glaucoma, emulsified oil in the anterior chamber or secondary angle closure.[65,66] Independent risk factors for developing silicone oil glaucoma include rubeosis iridis, aphakia, diabetes, and the use of highly purified silicone oil (5000 cSt) or its presence in the anterior chamber.

Silicone oil removal alone may be effective to lower IOP,[65] but is not always possible. The ability to control IOP medically is variable (30-78%)[65,67] with more invasive treatment often required. Where angle closure contributes, PLI may be attempted, but there is a lower success rate and need for multiple treatments,[68] compared with primary angle closure. Trabeculectomy and cycloablation have inferior outcomes to those reported for GDD insertion.[65] With an inferiorly placed shunt tube to minimize silicone oil passage into the bleb, Ishida *et al*.[69] have reported successful IOP control after 4 years in 70% of patients uncontrolled on medication alone. Although the failure rate was higher compared with eyes without silicone oil, it still appears to be a better option than either trabeculectomy or cycloablation.

### Glaucoma and penetrating keratoplasty

Glaucoma and corneal disease often coexist. Elevated IOP, particularly in aphakia or pseudophakia, may lead to bullous keratopathy. Similarly, PK for any reason may be complicated by elevated IOP in the postoperative period and is a common cause for graft failure. The risk of developing post-PK glaucoma may be related to the underlying condition requiring corneal surgery. The rate following Fuchs’ endothelial dystrophy and keratoconus is low compared with infective keratitis and corneal perforation.[70] In the acute perioperative period, IOP may be elevated because of retained viscoelastic, persistent inflammation or pre-existing glaucoma. In the longer term, IOP control may become difficult because of a steroid response, synechial angle closure or epithelial downgrowth.

Elevated IOP from long-term topical steroids postoperatively is problematic and may partly explain the difficulty with pressure control in patients with pre-existing glaucoma. Early withdrawal of topical steroids in such patients could aid IOP control, but increases the risk of graft rejection. An alternative strategy might involve substituting topical steroids with a non-steroidal anti-inflammatory anti-rejection agent without known IOP effects. Two prospective studies used cyclosporine A instead of a topical steroid in steroid responders following PK.[71,72] In both studies, there was a significant reduction in mean IOP following cyclosporine A treatment. Most patients maintained graft clarity though an increased risk of allograft rejection was reported. In cases where the trabecular meshwork is clearly visible on gonioscopy, LT may be an option.[73]

Trabeculectomy may be performed where there has been previous PK and glaucoma is uncontrolled despite medical therapy. Trabeculectomy alone may fail to control IOP in 75–90% of cases,[74,75] whereas intraoperative antimetabolite use improves outcome significantly.[75] Adverse outcome is more likely in patients who have had multiple PKs or if there is synechial angle closure.[74] While a trabeculectomy may threaten graft survival (5 year probability is 0.62),[76] failure to control IOP is likely to cause graft failure.[77]

Transscleral diode laser photocoagulation may be performed for refractory glaucoma following PK. In a series of 32 patients,[78] IOP was controlled in 72%, with 44% needing re-treatment during the 12-month follow-up. Importantly, no graft failure was observed.

In cases where severe corneal disease and poorly controlled glaucoma coexist, PK is destined to fail unless the IOP is controlled simultaneously. Trabeculectomy with antimetabolite combined with PK has a good chance to control IOP (91%) and maintain graft clarity (82%) in the short to medium term.[79] Less successful has been the combination of GDD with PK. Although IOP often is well controlled, graft failure is universal by 5 years and appears to be independent of anterior chamber[80] or pars plana tube placement.[81]

Postoperative glaucoma remains an issue for newer corneal grafting techniques. Elevated IOP is common after Descemet’s Stripping Endothelial Keratoplasty (DSEK) in patients with or without pre-existing glaucoma,[82] although no adverse effect on visual improvement was noted. Those with pre-existing glaucoma were more likely to need steroid reduction or topical glaucoma medication to control IOP, and prior trabeculectomy was a risk factor for post-DSEK trabeculectomy (prior trabeculectomy 19%, glaucoma 5%, no glaucoma 0.3%).

## Globe Injury and Glaucoma

Acute or delayed IOP rises may occur following ocular trauma. Early-onset IOP elevation may result from anterior segment inflammation, disruption of trabecular meshwork, hyphema or lens-related glaucoma. Angle recession, ghost cells, hemolysis, hemosiderin, lens-related glaucoma or retained intraocular foreign body are all potential causes of elevated IOP later on. The incidence of elevated IOP within 6 months of closed globe injury is approximately 3.4%.[83] Increased risk of developing raised IOP is seen in individuals presenting with hyphema, angle recession, lens dislocation, traumatic cataract, angle pigmentation, IOP > 21 mmHg at presentation, VA < 20/200 at presentation and increasing age. Successful management requires careful identification of contributing mechanisms and addressing them accordingly.

Angle recession is a major contributor to impaired IOP regulation following blunt ocular trauma. It is present in up to 100% of individuals with trauma-induced hyphema; an estimated 7–9% of these patients go on to develop angle recession glaucoma. Elevated IOP may occur because of collapse, atrophy and fibrosis of the trabecular meshwork as well as effects on the ciliary body itself. Therapy that increases outflow facility, such as miotics and laser trabeculoplasty,[84] are less effective than in POAG. Suppression of aqueous production should be more successful to lower IOP, but the efficacy of β-blockers, α-agonists or carbonic anhydrase inhibitors has not been studied specifically in these patients. Also, the response to prostaglandin analogues is yet to be studied.

As many patients with angle recession glaucoma have inadequate IOP control on medication alone, surgical treatment is often required. However, retrospective analysis has shown angle recession to be a risk factor for trabeculectomy failure.[85] Compared with POAG, successful trabeculectomy in angle recession is significantly lesser (74% vs. 43%) with most failures occurring within a few months of surgery. Superior outcomes have been achieved with trabeculectomy combined with intraoperative metabolite (77% IOP control)[86] whilst a study examining GDDs showed a low success rate comparable with trabeculectomy without antimetabolite.[87]

## Conclusion

Management of complex glaucoma has progressed on two fronts. Firstly, studies show the most appropriate ways to apply available therapies. Examples include the recently documented efficacy of prostaglandin analogues in uveitic glaucoma and the utility of LT in steroid-induced glaucoma. Secondly, novel treatments are emerging which offer hope in conditions where the outcome was previously invariably poor: anti-VEGF therapy for rubeotic glaucoma. Together, these advances allow clinicians to maximize outcomes for their patients with complex glaucoma.
